# ^19^F NMR reveals the conformational properties of free thrombin and its zymogen precursor prethrombin-2

**DOI:** 10.1074/jbc.RA120.013419

**Published:** 2020-05-01

**Authors:** Eliza A. Ruben, Prafull S. Gandhi, Zhiwei Chen, Sarah K. Koester, Gregory T. DeKoster, Carl Frieden, Enrico Di Cera

**Affiliations:** 1 Edward A. Doisy Department of Biochemistry and Molecular Biology, Saint Louis University School of Medicine, St. Louis, Missouri, USA; 2 Global Research, Novo Nordisk, Måløv, Denmark; 3 Department of Biochemistry and Molecular Biophysics, Washington University School of Medicine, St. Louis, Missouri, USA

**Keywords:** nuclear magnetic resonance (NMR), X-ray crystallography, thrombin, serine protease, protein conformation, blood coagulation, ligand binding, protein dynamics, zymogen

## Abstract

The conformational properties of trypsin-like proteases and their zymogen forms remain controversial because of a lack of sufficient information on their free forms. Specifically, it is unclear whether the free protease is zymogen-like and shifts to its mature form upon a ligand-induced fit or exists in multiple conformations in equilibrium from which the ligand selects the optimal fit via conformational selection. Here we report the results of ^19^F NMR measurements that reveal the conformational properties of a protease and its zymogen precursor in the free form. Using the trypsin-like, clotting protease thrombin as a relevant model system, we show that its conformation is quite different from that of its direct zymogen precursor prethrombin-2 and more similar to that of its fully active Na^+^-bound form. The results cast doubts on recent hypotheses that free thrombin is zymogen-like and transitions to protease-like forms upon ligand binding. Rather, they validate the scenario emerged from previous findings of X-ray crystallography and rapid kinetics supporting a pre-existing equilibrium between open (E) and closed (E*) forms of the active site. In this scenario, prethrombin-2 is more dynamic and exists predominantly in the E* form, whereas thrombin is more rigid and exists predominantly in the E form. Ligand binding to thrombin takes place exclusively in the E form without significant changes in the overall conformation. In summary, these results disclose the structural architecture of the free forms of thrombin and prethrombin-2, consistent with an E*–E equilibrium and providing no evidence that free thrombin is zymogen-like.

## Introduction

The trypsin fold defines the structural architecture of a mature “active” protease and its immature “inactive” zymogen precursor and has been studied in considerable detail, both functionally and structurally (1–3). The zymogen-to-protease transition is described by the Huber–Bode mechanism (4) and involves a proteolytic cleavage at a conserved Arg residue in the so-called activation domain, followed by insertion of the new N terminus into the protein core and folding of the active site. The mechanism was originally assumed to generate a fully functional protease from its inactive zymogen precursor, but this view proved too simplistic when structures of trypsin-like proteases started to accumulate in the Protein Data Bank (PDB).

The trypsin fold has long been assumed to be mostly “rigid” (5–8). However, the entire “west wall” of the active site defined by the amino acid segment at positions 215–217 adopts different conformations that open and close access to the primary specificity pocket at the bottom of the active-site region. This conformational plasticity is of functional significance (9). In the open conformation (E), the active site enables ligand binding with high affinity. In the closed conformation (E*), no binding is possible. The active site is occluded by collapse of the side chain of Trp^215^ and a shift of the backbone of the segment at positions 215–217. The aperture leading to the primary specificity pocket and defined by the Cα–Cα distance of residues Gly^193^ and Gly^216^ changes from 12 Å in the E form to only 8.1 Å in the E* form (10). In the D216G mutant of αI-tryptase (11), chymotrypsinogen (12), and the thrombin precursor prethrombin-2 (13), both the E and E* forms are detected in the same crystal. Independent support of alternative conformations for the free form of protease and zymogen comes from rapid kinetics studies of ligand binding to the active site. The mechanism of recognition for small tripeptides is consistent with a pre-existing E*–E equilibrium of conformational selection rather than induced fit (10, 14). The E* form prevails in the zymogen and gradually shifts to the E form during transition to the mature protease (15). In fact, the E*–E equilibrium complements the Huber–Bode mechanism and contributes to organization of the active site for efficient binding and catalysis (16).

Although evidence of the E*–E equilibrium from the current structural database (9, 17, 18) and rapid kinetics (10, 14, 15, 17) is solid, direct proof that such conformational plasticity exists in solution remains elusive. Structural features revealed by X-ray may be biased by crystal packing or by the extreme solution conditions often necessary to achieve crystallization. Kinetic experiments provide unequivocal evidence of conformational selection only when certain conditions are met for the observed relaxation rates (19). Previous NMR studies on the clotting protease thrombin in different bound states (20) have identified a progressive rigidification of the enzyme upon ligation. From these studies, Huntington (21) and Krishnaswamy and co-workers (22) have concluded that thrombin is inherently plastic and shuttles within an ensemble of conformations that are disordered and zymogen-like when free but rigid and protease-like when bound (21, 22). However, this ensemble view of thrombin (20–22) remains a speculation largely inconsistent with existing X-ray (9, 17, 18) and rapid kinetics (10, 14, 15, 17) data and lacks validation from NMR studies of free thrombin or of the zymogen precursor prethrombin-2. Whether free thrombin is zymogen-like and switches to the mature conformation by induced fit or pre-exists in alternative conformations from which the ligand selects the optimal fit can only be established by studies of the free form. Recent NMR measurements by the Komives group (23) have targeted the “apo-form” of thrombin using the S195M mutant and compared the dynamics with those of thrombin bound at the active site (24). However, these studies have been carried out in the presence of Na^+^ and therefore describe the dynamics of ligation of the fully active Na^+^-bound form of thrombin rather than its free form.

In this study, we investigate for the first time the conformation of the protease thrombin and its zymogen precursor prethrombin-2 in the free form using ^19^F NMR. We labeled all nine Trp residues in thrombin and prethrombin-2 to interrogate the conformational properties of the protein in the absence of any added ligand and Na^+^. ^19^F NMR has been used for a number of biologically important systems (25–29) and is ideally suited for studying the Trp residues of thrombin that are known to report on changes linked to the E*–E equilibrium, the zymogen-to-protease conversion, and ligand binding (14, 15, 30).

## Results

### ^19^F labeling

**Figure 1.**
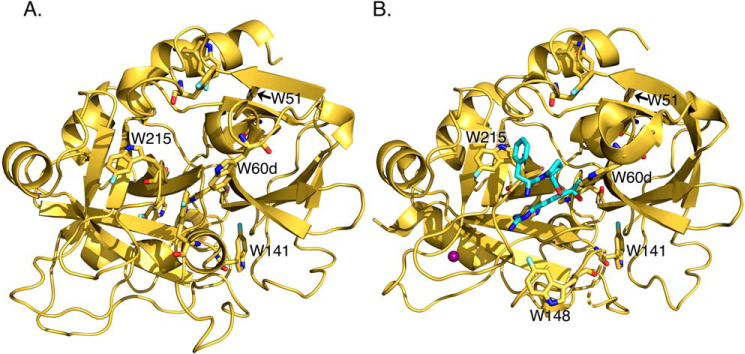
**Crystal structures of ^19^F-labeled prethrombin-2 (*A*) and PPACK (*cyan sticks*) inhibited thrombin in the Na^+^-bound (*purple ball*) form (*B*) with the side chains of the nine Trp residues of the protein shown as *sticks*.** Residues Trp^51^ and Trp^215^ feature characteristic NMR resonances and dynamics (see Figs. 4 and 5) and are indicated, along with residues Trp^141^, Trp^148^, and Trp^60d^. The ^19^F label on the 5 position of the indole ring is clearly visible for all Trp residues (see also Fig. 2). The two structures are similar to unlabeled prethrombin-2 (13) (RMSD = 0.33 Å) and PPACK-bound thrombin (8, 37) (RMSD = 0.41 Å), proving that labeling introduced no bias in the fold. Details of the structures are given in Table 1.

**Figure 2.**
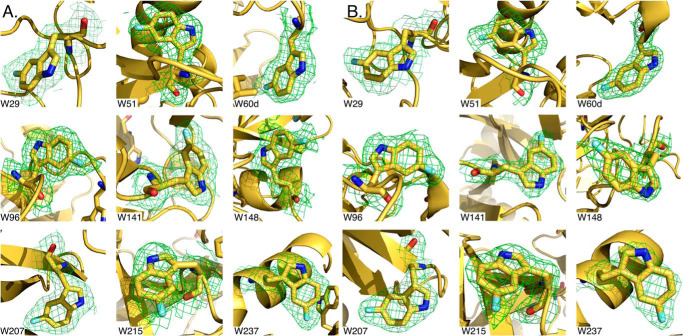
**Details of the ^19^F-labeled Trp residues of prethrombin-2 (*A*) and thrombin (*B*), taken from the crystal structures shown in Fig. 1 (see also Table 1).** Extra density on the 5 position of the indole side chain is clearly detected for all nine Trp residues, demonstrating that labeling was uniform. The electron density 2*F*_o_ − *F*_c_ map (*green mesh*) is contoured at 1 σ.

We labeled all Trp residues of thrombin and prethrombin-2 at the 5 position of the indole ring with ^19^F and solved the X-ray structures at 2.3 and 2.1 Å resolution, respectively (Table 1). The nine Trp residues are distributed over the entire surface and function as effective reporters of the conformational state of the protein. The structures show all Trp residues correctly labeled without significant perturbation of the overall architecture (Fig. 1). Extra density detected at the 5 position of the indole ring in all cases supports uniform labeling of the reagents used for NMR studies (Fig. 2). The ^19^F-labeled prethrombin-2 structure features a conformation of the active site similar to that of WT (13) (RMSD = 0.33 Å). The ^19^F-labeled thrombin structure bound to the active site inhibitor PPACK and Na^+^ is very similar to the unlabeled complex (8, 37) (RMSD = 0.41 Å).

**Table 1:** **Crystallographic data for ^19^F-labeled prethrombin-2 and PPACK-inhibited thrombin**

**PDB entry**	6V5T	6V64
Buffer/salt	0.1 m HEPES, pH 7.0	0.2 m sodium/potassium tartrate, pH 7.5
PEG	8000 (25%)	3350 (14%)
**Data collection**		
Wavelength (Å)	1.54	1.54
Space group	P2_1_	P2_1_2_1_2
Unit cell dimensions (Å)	*a* = 44.5, *b* = 58.9, *c* = 52.4, β = 98.4	*a* = 61.9, *b* = 86.6, *c* = 50.5
Molecules/asymmetric unit	1	1
Resolution range (Å)	40–2.1	40–2.3
Observations	79,521	62,626
Unique observations	15,696	12,020
Completeness (%)	99.3 (97.0)	94.9 (84.5)
*R*_sym_ (%)	7.3 (55.9)	11.5 (33.4)
*I*/σ(*I*)	18.0 (2.4)	11.7 (2.4)
**Refinement**		
Resolution (Å)	40–2.1	40–2.3
*R*_cryst_, *R*_free_	0.177, 0.230	0.197, 0.277
Reflections (working/test)	14,911/772	11,333/588
Protein atoms	2,356	2,283
Solvent molecules	103	108
PPACK		1
Na^+^		2
RMSD bond lengths (Å)*^a^*	0.008	0.010
RMSD angles (°)*^a^*	1.5	1.8
RMSD ΔB (Å^2^) (mm/ms/ss)*^b^*	3.21/2.98/3.50	2.04/2.20/2.08
Protein	41.5	43.6
Solvent	42.6	40.6
PPACK		32.3
Na^+^		33.8
Ramachandran plot (%)		
Most favored	95.0	95.0
Generously allowed	5.0	5.0
Disallowed	0.0	0.0

*^a^* RMSD from ideal bond lengths and angles and RMSD in B-factors of bonded atoms.

*^b^* mm, main chain–main chain; ms, main chain–side chain; ss, side chain–side chain.

### ^19^F NMR measurements

**Figure 3.**
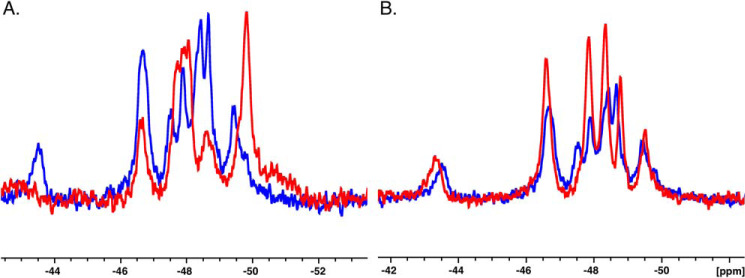
**Overlay of 1D NMR spectra between prethrombin-2 and thrombin (*A*) and between free thrombin and thrombin bound to Na^+^ (*B*).** The spectra show how free thrombin is more similar to its Na^+^-bound form than the zymogen precursor prethrombin-2. The difference is particularly noticeable in the number of separate peaks detected for zymogen and protease, consistent with a more rigid structure for the latter.

Having established uniform ^19^F labeling by X-ray crystallography, we proceeded to collect 1D ^19^F NMR spectra for thrombin and prethrombin-2 in the free form, devoid of ligands bound to the active site or Na^+^. These conditions have not been explored in previous studies (20, 23, 24) but are essential to determine the intrinsic properties of the protein in the free form. Overall, the 1D ^19^F spectra show seven well-dispersed peaks for thrombin and only four for prethrombin-2 (Fig. 3*A* and Table 2). The most striking differences between zymogen and protease are the resonance at −43.5 ppm for thrombin not seen in prethrombin-2, and the range between −47.2 and −49.0 ppm where thrombin shows four distinct peaks, but prethrombin-2 features only two, one large and broad (−47.9 ppm) and the other smaller (−48.6 ppm). A sharp resonance observed in prethrombin-2 around −49.8 ppm is replaced by a smaller one in thrombin, slightly shifted to −49.4 ppm. The presence of well-defined and separable peaks in thrombin as opposed to prethrombin-2 suggests that most of the Trp residues in the zymogen experience a similar chemical environment. However, this conclusion is not supported by the crystal structure (Fig. 1) where some Trp residues are exposed to solvent (Trp^60d^, Trp^148^, and Trp^215^) and others are more buried (Trp^51^). An alternative explanation is that Trp residues in prethrombin-2 exchange among multiple conformations leading to broad, overlapping linewidths. Hence, thrombin likely explores a smaller conformational space and is intrinsically more rigid than its zymogen precursor prethrombin-2. The observation points out significant differences between protease and zymogen in the free form and does not support recent claims of free thrombin being zymogen-like (20–22, 24). In fact, free thrombin is way more similar to its Na^+^-bound form (Fig. 3*B*) than its zymogen precursor prethrombin-2 (Fig. 3*A*). Rapid kinetics studies suggest that a significant fraction of the free enzyme exists in the E form (10). Structural studies document almost complete overlap between the free and bound E forms (37, 38). The addition of Na^+^ is known to boost the catalytic activity of the enzyme (39) and to rigidify the structure (20, 40). The ^19^F NMR spectra in Fig. 3*B* show that binding of Na^+^ sharpens and better separates the peaks of free thrombin and removes the peak at −47.9 ppm. We conclude that free thrombin is not zymogen-like. Rather, it is quite distinct from its zymogen precursor prethrombin-2 and already contains features of its more rigid, Na^+^-bound form as predicted by a mechanism of conformational selection (10, 19, 41, 42).

**Table 2:** **^19^F chemical shifts (ppm) of Trp residue** ND, not determined.

	Thrombin	Prethrombin-2
Trp^29^	ND	ND
Trp^51^	−46.7	−46.7
Trp^60d^	−48.5, −48.7	−47.9
Trp^96^	−47.9	−47.9
Trp^141^	−43.5	−47.9
Trp^148^	−48.5	−47.9
Trp^207^	ND	−47.9
Trp^215^	−47.5	−47.9, −49.8
Trp^237^	−49.4	−48.6

### Resonance assignment

**Figure 4.**
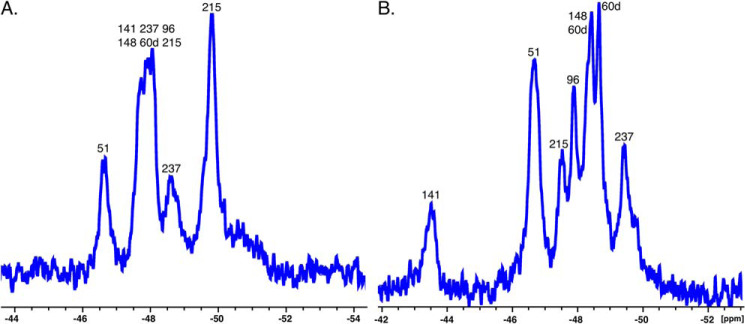
**1D NMR spectra of prethrombin-2 (*A*) and thrombin (*B*) with peaks assigned from single site replacement of Trp with Phe (see also Fig. 5).** The substitution does not change the functional properties and specificity of thrombin (30). *A*, assignment of individual Trp residues in prethrombin-2 often did not result in selective perturbation of peaks in the spectrum (Fig. 3*A*). The W215F replacement affected both the large peak around −47.9 ppm and the peak at −49.8 ppm (Fig. 5). Mutations of Trp^60d^, Trp^96^, Trp^141^, Trp^148^, Trp^207^, and Trp^215^ perturbed the peak at −47.9 ppm, and those of Trp^51^ and Trp^237^ affected the peaks at −46.7 and −48.6 ppm, respectively. Trp^29^ and Trp^207^ could not be assigned. *B*, clusters are less pronounced than in prethrombin-2. Mutations of Trp^141^, Trp^51^, Trp^215^, Trp^96^, and Trp^237^ mapped to peaks at −43.5, −46.7, −47.5, −47.9, and −49.4 ppm, respectively. Trp^51^ mapped to the same resonance position as in prethrombin-2, but Trp^237^ was shifted upfield. Of the remaining four Trp residues, Trp^29^ and Trp^207^ could not be assigned, and Trp^148^ and Trp^60d^ clustered in the peaks in the range of −47 to −48 ppm and could not be separated.

**Figure 5.**
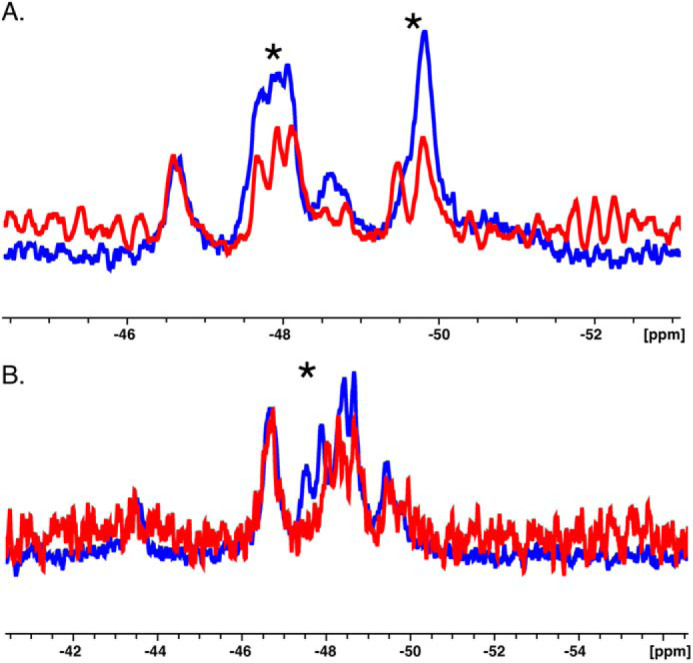
**Overlay of 1D NMR spectra between WT (*blue*) and mutant W215F (*red*) for prethrombin-2 (*A*) and thrombin (*B*).** *Asterisks* indicate the region of the spectrum perturbed by the single-site replacement.

Given the differences between zymogen and protease in the free form, we turned our attention to the specific Trp residues responsible for the observed changes. Assignments of the nine Trp residues in both thrombin and prethrombin-2 (Fig. 4, *A* and *B*) were made from spectra for which each individual Trp residue was replaced by Phe. The substitution is inconsequential on the catalytic properties and specificity of thrombin and has been used to identify the fluorophores responsible for Na^+^ binding to the enzyme (30). Assignment of individual Trp residues was often complicated by the lack of selective perturbation of peaks in the WT spectrum. In the case of prethrombin-2, the W215F substitution affects both the large peak around −47.9 ppm and the peak at −49.8 ppm, almost 2 ppm apart (Fig. 5*A*). This suggests that Trp^215^ exists in alternative conformations that exchange very slowly. Mutations of Trp^60d^, Trp^96^, Trp^141^, Trp^148^, Trp^207^, and Trp^215^ result in perturbation of the peak at −47.9 ppm (Fig. 4*A*). Residues Trp^51^ and Trp^237^ map to the peaks at −46.7 and −48.6 ppm, respectively, but Trp^29^ and Trp^207^ could not be assigned. In the case of thrombin (Fig. 4*B*), clustering is less pronounced, and residues Trp^141^, Trp^51^, Trp^215^, Trp^96^, and Trp^237^ could be assigned to resonances at −43.5, −46.7, −47.5, −47.9, and −49.4 ppm, respectively (see Fig. 5*B* for W215F). The relative solvent exposure of these residues is consistent with the crystal structure (8, 43) (see also Fig. 1*B*). Trp^51^ maps to the same resonance position as in prethrombin-2 (Fig. 4*A*), but Trp^237^ is shifted upfield. Of the remaining four Trp residues, Trp^29^ and Trp^207^ could not be assigned, whereas Trp^148^ and Trp^60d^ cluster in the peaks within the range of −47 to −48 ppm and could not be separated, suggesting similar solvent exposure as seen in the crystal structure (8, 43) (see also Fig. 1*B*). We conclude that prethrombin-2 is more dynamic than thrombin, with most of its Trp residues in slow exchange between alternative conformations and experiencing an environment that changes significantly during the conversion to thrombin.

### Residue dynamics

**Figure 6.**
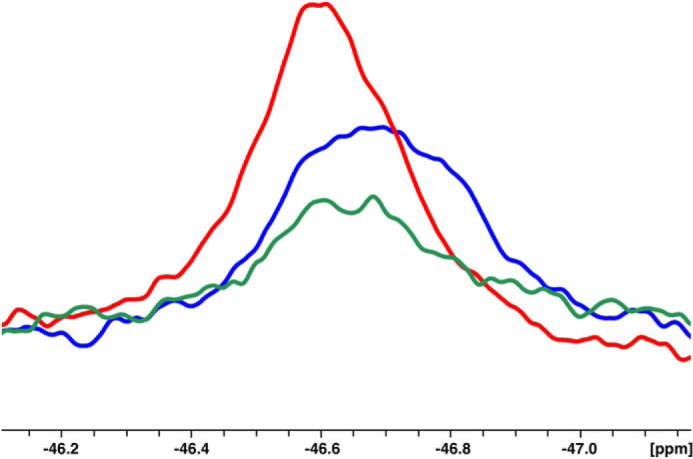
**Overlay of resonances for Trp^51^ in prethrombin-2, free thrombin, and thrombin bound to Na^+^ indicating distinct line broadening of Trp^51^.**

**Figure 7.**
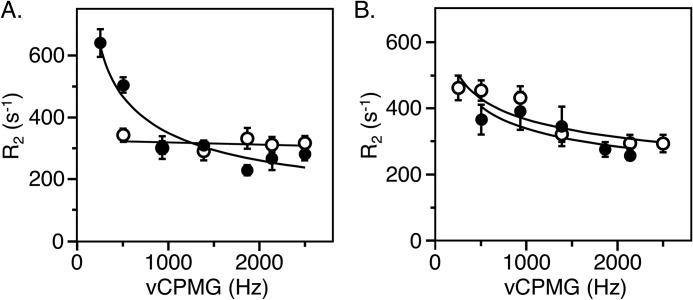
**CPMG relaxation dispersion profiles in the fast time-scale regime of residues Trp^51^ (*filled circles*) and Trp^215^ (*open circles*) in prethrombin-2 (*A*) and thrombin (*B*).**

**Figure 8.**
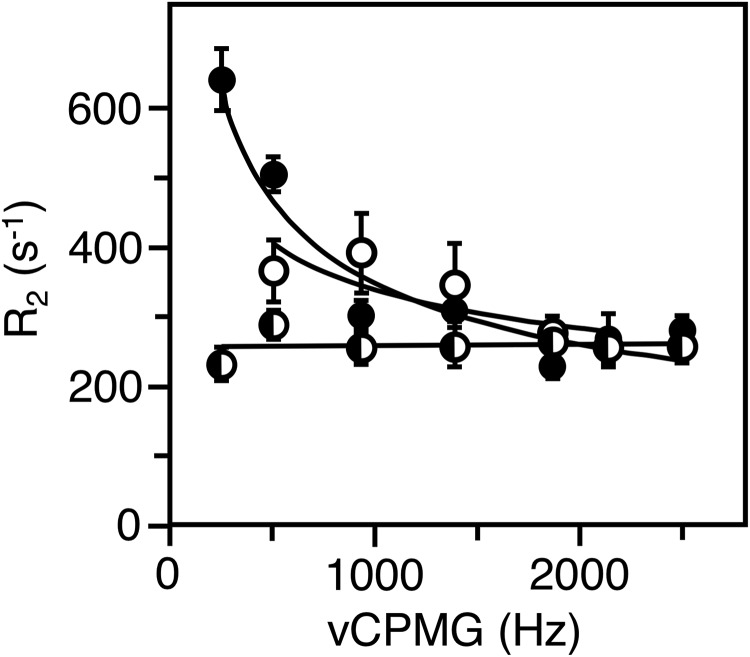
**Field-dependent ^19^F CPMG relaxation dispersion data for Trp^51^ in prethrombin-2 (*filled circles*), free thrombin (*open circles*), or thrombin bound to Na^+^ (*half-filled circles*).**

**Figure 9.**
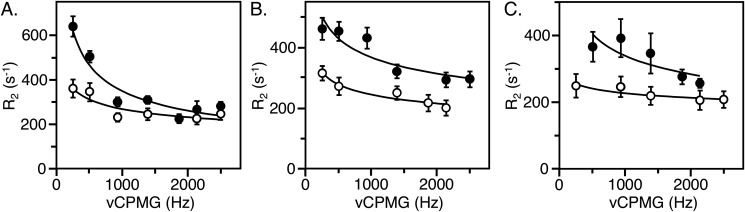
**Field-dependent CPMG relaxation dispersion profiles in the fast time-scale regime.** *Filled circles* represent transverse relaxation rates (*R*_2_) as a function of υcpmg acquired at 658.780 MHz, and *open circles* represent transverse relaxation rates (*R*_2_) as a function of υcpmg acquired at 564.686 MHz. The data were fit to a two-state model in the fast-exchange regime yielding the following: *A*, residue Trp^51^ in prethrombin-2, *k*_ex_ = 2,970 ± 20 s^−1^, *R*_2_^0^ (564.686 MHz) = 204 ± 1 s^−1^, *R*_2_^0^ (658.780 MHz) = 260 ± 0.3 s^−1^. *B*, residue Trp^215^ in thrombin. *k*_ex_ = 7,980 ± 90 s^−1^, *R*_2_^0^ (564.686 MHz) = 160 ± 1 s^−1^, *R*_2_^0^ (658.780 MHz) = 258 ± 1 s^−1^. *C*, residue Trp^51^ in thrombin, *k*_ex_ = 19,000 ± 1,000 s^−1^, *R*_2_^0^ (564.686 MHz) = 100 ± 10 s^−1^, *R*_2_^0^ (658.780 MHz) = 140 ± 10 s^−1^.

Individual resonances could be assigned to residues Trp^51^ and Trp^215^ in thrombin and Trp^51^ in prethrombin-2. These residues were investigated further by measurements of T1, T2, and CPMG relaxation dispersion to gain insight into their range of motions. Trp^51^ is positioned 33 Å away from the Na^+^ binding site and 22 Å away from the catalytic Ser^195^ (Fig. 1). The peak for Trp^51^ has the same resonance position at −46.6 ppm in both thrombin and prethrombin-2 (Fig. 4) and broadens from 0.27 to 0.37 Hz relative to thrombin bound to Na^+^ (Fig. 6), suggesting the presence of multiple conformations. Indeed, the peak shows a distinct relaxation dispersion profile indicative of conformational exchange, especially in prethrombin-2 (Figs. 7 and 8). Collection at a second field of 600 MHz allowed relaxation dispersion curves at both fields to be fit to a two-state model in the fast-exchange regime (44) with *k*_ex_ = 19,000 ± 1,000 s^−1^ in thrombin and *k*_ex_ = 2,970 ± 20 s^−1^ in prethrombin-2 (Fig. 9). The intrinsic dynamics of Trp^51^ indicate faster exchange in thrombin than prethrombin-2. Interestingly, the exchange in thrombin is completely abrogated upon Na^+^ binding (Fig. 8), suggesting rigidification of a residue located 33 Å away. The slower exchange at Trp^51^ observed in prethrombin-2 is indicative of the presence of more large-scale motions compared with thrombin. These findings add complexity to the scenario emerged from the X-ray structural database where the conformation of Trp^51^ is essentially the same in prethrombin-2 (13) and thrombin free or bound to ligands (37). We conclude that Na^+^ binding has long-range effects on the structure of thrombin and that Trp^51^ is allosterically coupled to regions affected by the zymogen to protease conversion, as well as Na^+^ binding, thereby establishing a new allosteric pathway of communication within the protein that affects widely separated residues.

Residue Trp^215^ defines the P3 site of recognition for substrate binding to the active site (8) and exists in different conformations that open and close access to the active site according to the X-ray structural database (9, 18). The role of the indole side chain of Trp^215^ in the E*–E equilibrium has been tested by rapid kinetics with the W215A mutation and found not to be responsible for the opening and closing of access to the primary specificity pocket (14). Specifically, the W215A mutant binds ligands at the active site with a mechanism of conformational selection as WT, proving that removal of the side chain of Trp^215^ does not equalize access to the active site between the E* and E forms. The role of the side chain of Trp^215^ is to keep the active site open and slow down the E → E* conversion by establishing an interaction with the benzene ring of Phe^227^ (14). Once this hydrophobic interaction is disrupted, closure of the active site in the E* form is faster and results in reduced catalytic activity (45, 46). The rate of exchange between E* and E in thrombin and prethrombin-2 becomes of interest. Trp^215^ maps to a single peak at −47.5 ppm in thrombin (Figs. 4*B* and 5*B*). In prethrombin-2, Trp^215^ maps with other residues to the broad peak in the range of −47 to −48 ppm and also to a unique peak close to −49.8 ppm (Figs. 4*A* and 5*A*). The presence of two peaks separated by a large chemical shift indicates that Trp^215^ features two distinct conformations that exchange very slowly or not at all in prethrombin-2 (Fig. 7). The dynamic profile changes upon transition to thrombin and supports fast exchange between two states (Fig. 7). Measurements at different field strength (Fig. 9) yield a rate of exchange *k*_ex_ = 7,980 ± 90 s^−1^. Although the structural database documents a similar behavior for Trp^215^ in zymogen and protease with regard to the E* and E forms controlling access to the primary specificity pocket (9, 18), the dynamics of Trp^215^ are consistent with an exchange considerably faster in thrombin than prethrombin-2.

## Discussion

Our understanding of the conformational nature of trypsin-like proteases and their zymogens has been deeply influenced by the celebrated Huber–Bode mechanism of zymogen activation (4). Activity is assumed to result from proteolytic cleavage of a conserved Arg residue in the activation domain followed by an ionic interaction that is established between the new N terminus of Ile^16^ and the side chain of the highly conserved Asp^194^. The newly formed H-bond between Ile^16^ and Asp^194^ organizes the oxyanion hole around Gly^193^ and the catalytic Ser^195^ and the primary specificity pocket around Asp^189^. This transition, however, is neither necessary nor sufficient to generate a fully active protease. Activity can be triggered by alternative mechanisms. Single-chain tissue-type plasminogen activator features catalytic activity by establishing an intramolecular H-bond that produces the same structural transitions as the Huber–Bode mechanism (47). A similar strategy is used by the plasminogen activator in the saliva of *Desmodus rotundus* (48). Bacteria have evolved proteins like streptokinase (49) and staphylocoagulase (50) that can activate the host fibrinolytic and coagulation cascades without proteolytic cleavage of their target zymogens plasminogen or prothrombin. An entire class of zymogen activator peptides mimicking streptokinase and staphylocoagulase has been developed by phage display (51). The Huber–Bode mechanism also appears not to be sufficient for protease function. A pre-existing equilibrium between closed (E*) and open (E) conformations of the active site controls the onset of substrate binding and catalysis (15). In the E* form, substrate cannot bind to the active site, and catalysis is impeded. Importantly, the balance between E* and E changes between zymogen and protease, with the E* form predominating in the former (15, 16) and presaging little overlap between the free forms of protease and its zymogen precursor.

The results reported in this study offer a view of the structural architecture of thrombin and prethrombin-2 in the free form that is entirely consistent with the E*–E equilibrium. We find no evidence that free thrombin is zymogen-like, as speculated in previous functional (20–22) and computational (52) studies. Labeling all nine Trp residues of the protein for ^19^F NMR measurements show that free thrombin is quite different from prethrombin-2 and more similar to its Na^+^-bound form, in agreement with the structural differences between E* and E (17, 37), the predominance of E* for prethrombin-2 and of E for thrombin (10, 17, 19), and the fact that E form changes little upon ligand binding (17, 38).

Our ^19^F NMR data provide information on the dynamics of critical Trp residues of the protein. Most of these residues cannot be assigned in the 1D ^19^F NMR spectrum of prethrombin-2 because of overlap of linewidths. Trp^51^ maps to a single peak and features rapid exchange between alternative conformations in both prethrombin-2 and thrombin. Trp^215^ maps to two widely separated peaks, indicating a very slow exchange between alternative conformations. When prethrombin-2 transitions to thrombin upon activation, the overall structure becomes less dynamic, with several individual peaks in the 1D ^19^F NMR spectrum that can be assigned to specific Trp residues. Unlike Trp^51^, residue Trp^215^ features distinct dynamics from prethrombin-2 and exchanges rapidly between alternative conformations with *k*_ex_ = 7,980 ± 90 s^−1^, which is significantly faster than the rate for the E*–E exchange detected by rapid kinetics (10, 15). Although the dynamic nature of Trp^215^ (Fig. 5*B*) is consistent with alternative conformations documented by the structural database (9, 17, 18), its fast time scale of exchange points to events that eventually do not influence access to the active site as documented in the E*–E equilibrium. Indeed, removal of the indole of Trp^215^ with the W215A substitution changes little the E*–E distribution compared with WT thrombin (14, 53). Other features of the protein, like movement of the backbone of the 215–217 segment, may be responsible for the E*–E equilibrium (10) and should be investigated further by NMR of the free form to extend the work reported in this study.

## Materials and methods

### Reagents

Prethrombin-2 cloned into a pet28 *Escherichia coli* expression vector was transformed into the BL21DE3 *E. coli* strain. 50-ml starter cultures in LB supplemented with ampicillin were grown for up to 16 h at 37 °C in an orbital shaker rotating at 225 rpm. Starter cultures were diluted 1:50 in LB, also supplemented with ampicillin, and grown for a further 3–4 h until *A*_600_ > 1.0 was reached. The growth culture was spun down at 4,000 rpm for 20 min. The pellet was then dissolved in minimal medium for incorporation of 5-F-Trp and grown for an additional 2 h before recombinant protein expression was induced by adding 1 mm isopropyl β-d-thiogalactopyranoside and growing at 25 °C overnight. The formulation for 5-F-Trp minimal media reads as 50 mm Na_2_HPO_4_, 25 mm KH_2_PO_4_, 20 mm NH_4_Cl, 100 μg/ml ampicillin, 0.25 mg/liter 5-F-Trp, and 0.4% w/v d-glucose.

For inclusion body purification and refolding, the cells were pelleted by centrifugation at 4,000 rpm for 25 min and resuspended in 50 mm Tris, pH 7.4, 20 mm EDTA, 1 mm DTT, and 1% Triton X-100. The cells were lysed using an Avestin C3 emulsified or sonicator. Inclusion bodies were separated by centrifugation at 10,000 rpm for 15 min. Inclusion bodies were washed sequentially in 50 mm Tris, pH 7.4, 20 mm EDTA, and 1 m NaCl followed by 50 mm Tris, pH 7.4, and 20 mm EDTA. Inclusion bodies were solubilized in 40 ml of 7 m guanidine HCl and homogenized using a Dounce homogenizer. Inclusion bodies were centrifuged at 10,000 rpm for 10 min to remove insoluble material. Inclusion bodies were refolded by dilution into 50× excess of 50 mm Tris, 500 mm NaCl, 1 mm EDTA, 10% glycerol, 600 mm Arg, 0.2% Brij58, 1 mm l-Cys, pH 8.3. Inclusion bodies were incubated at 25 °C overnight.

Properly folded prethrombin-2 was purified using a heparin-affinity column and buffer-exchanged through a size-exclusion column or 10-kDa Centricon into a final NMR buffer formulation of 20 mm Tris, 700 mm NaCl, or 700 mm choline chloride, 50 mm Arg, and 10% trehalose. Thrombin was generated from prethrombin-2 using ecarin and exchanged into the same NMR buffer. Protein was concentrated to ∼5 mg/ml and spiked with 10% D_2_O for ^19^F NMR data collection.

All constructs for NMR data collection contained neutralization of the active-site Ser^195^ with the S195A mutation to prevent autocatalytic degradation and enable collection of relaxation dispersion data over a period of days.

### ^19^F NMR studies

1D ^19^F NMR measurements were carried out at 658.780 MHz, 25 °C using a ^19^F QCI cryoprobe (City University of New York Advanced Science Research Center Biomolecular NMR Facility) and a 5-mm PFG quadruple resonance inverse detection cryoprobe (Saint Louis University High Resolution NMR Facility). No differences with 1D ^19^F NMR spectra were observed between the probes. Relaxation dispersion NMR experiments were carried out at 658.780 MHz, 25 °C (City University of New York Advanced Science Research Center Biomolecular NMR Facility) with a ^19^F QCI cryoprobe and at a second field strength of 564.686 MHz with a TCI (H/F-CN-D) cryogenic probe (Wisconsin). All spectra were referenced to TFA. Typical 1D ^19^F acquisition parameters were a 20,000-Hz sweep width (42.5 ppm), 0.35-s acquisition time, 5-s relaxation delay time, and 5.0-μs 90° pulse length. Spectra were processed with a 20-Hz exponential line broadening using topspin.

Longitudinal (T1) and transverse (T2) ^19^F relaxation measurements were determined using classic 1D inversion recovery (T1) and the Carr–Purcell–Meiboom–Gill (CPMG) spin echo pulse sequence (T2) (31, 32). ^19^F T1 inversion recovery experiments were acquired at a series of variable delay times (0.0625, 0.125, 0.25, 0.5, 1.0, 2.0, 4.0, and 8.0 s) and a relaxation delay of 7 s. ^19^F CPMG experiments consisted of a 90*x* − [τ_cp_ − 180*y* − τ_cp_] *n* pulse train acquired with a series of spin-echo evolution times (*e.g.* 8 points ranging between 0.5 and 128 ms). Longitudinal and transverse relaxation times were computed by fitting plots of ^19^F signal intensity for a given 5-F-Trp residue or peak as a function of variable decay (T1) or spin-echo times (T2). ^19^F CPMG relaxation dispersion experiments in which the transverse relaxation rate, *R*_2_, is determined as the function of the delay between 180° pulses (2τ_cp_) were acquired across a series of τ_cp_ values (*e.g.* 50–500 μs) and plotted against CPMG frequency (29). All measurements were collected in duplicate.

### X-ray studies

Crystallization of ^19^F-labeled, WT prethrombin-2, and thrombin bound to the active site inhibitor H-d-Phe-Pro-Arg-CH_2_Cl (PPACK) was achieved at 25 °C by the vapor diffusion technique, using an Art Robbins Instruments Phoenix^TM^ liquid handing robot with 6–10 mg/ml protein (0.3 μl) mixed with an equal volume reservoir solution. Optimization of crystal growth was achieved by the hanging-drop vapor-diffusion method mixing 3 μl of protein with equal volumes of reservoir solution (see Table 1). The crystals were grown in 1 week at 25 °C and frozen with 25% glycerol from the original mother liquor. X-ray diffraction data were collected at 100 K with a home source (Rigaku 1.2 kw MMX007 generator with VHF optics) Rigaku Raxis IV^2+^ detector and were indexed, integrated, and scaled with the HKL2000 software package (33). Structures were solved by molecular replacement using PHASER from the CCP4 suite (34) and the structures of human prethrombin-2 mutant S195A (PDB code 3SQH) and human thrombin in complex with PPACK (PDB code 1PPB) as starting models. Refinement and electron density generation were performed with REFMAC5 from the CCP4 suite. 5% of the reflections were randomly selected as a test set for cross-validation. Model building and analysis were carried out using COOT (35). In the final refinement stage, TLS tensors modeling rigid-body anisotropic temperature factors were calculated and applied to the model for the ^19^F-labeled thrombin bound to PPACK. Ramachandran plots were calculated using PROCHECK (36). The statistics for data collection and refinement are summarized in Table 1.

## Data availability

Atomic coordinates and structure factors for the two structures reported in the manuscript have been deposited in the PDB (accession code 6V5T for ^19^F-labeled prethrombin-2 and accession code 6V64 for ^19^F-labeled thrombin bound to PPACK). All other data described in the manuscript are contained within the manuscript.
